# A Nanocomposite with Extracellular Vesicles from *Lactobacillus paracasei* as a Bioinspired Nanoantibiotic Targeting *Staphylococcus aureus*

**DOI:** 10.3390/pharmaceutics14112273

**Published:** 2022-10-24

**Authors:** Atanu Naskar, Hyejin Cho, Kwang-sun Kim

**Affiliations:** Department of Chemistry and Chemistry Institute for Functional Materials, Pusan National University, Busan 46241, Korea

**Keywords:** extracellular vesicles, *Staphylococcus aureus*, nanocomposite, nanoantibiotic, biocompatibility, biomimetic material, *Lactobacillus paracasei*

## Abstract

The utilization of biomimetic materials that merge functional nanoparticles (NPs) with a cell-derived nanosized membrane is a state-of-the-art approach to harnessing cellular properties for biomedical applications. However, the development of biocompatible and species-selective biomimetic agents against hazardous pathogens threatening human health is still in its early stages. Herein, we report the synthesis and functional analysis of a novel nanoplatform in which a PEGylated MoS_2_-ZnO (MZ) nanocomposite was cloaked with a generally regarded as safe (GRAS)-grade *Lactobacillus paracasei*-derived extracellular vesicle (LPEV) for MZ-LPEV nanocomposite and evaluated its activity against *Staphylococcus aureus*. The MZ nanocomposite was characterized via X-ray diffraction, transmission electron microscopy, and X-ray photoelectron spectroscopy. The coating of MZ with LPEV was confirmed through nanoparticle tracking analysis and zeta potential measurements. MZ-LPEV exhibited 5- to 20-fold higher antibacterial activity than that of ZO NPs and MZ nanocomposite against *S. aureus*. Reactive oxygen species (ROS) production and bacterial membrane disruption were confirmed as antibacterial mechanisms of MZ-LPEV. Finally, MZ-LPEV exhibited enhanced biocompatibility and selectivity for *S. aureus*. All our results showed that LPEV could be utilized for developing synergistic nanoantibiotics against *S. aureus*.

## 1. Introduction

*Staphylococcus* is a large genus of Gram-positive bacteria that can colonize human tissue asymptomatically but may also cause simple skin infections or even lead to life-threatening illnesses [1]. Within this genus, *Staphylococcus aureus* is the leading life-threatening environmental infection owing to its ability to both effective colonization on environmental surfaces, air, skin, and mucous membranes and rapid transmission ability to the community [2,3,4,5]. The longitudinal association between persistent methicillin-resistant *S. aureus* (MRSA) colonization, environmental contamination, and recurrent human infections result in epidemics within various hospital settings and the rapid development of antibiotic resistance [6]. The currently available therapeutics or remediations for fighting *S. aureus* infections are limited [7]. Therefore, novel environmental remediation strategies are urgently needed.

Nanomaterials have attracted considerable interest due to their potential application in various domains, including healthcare. Since they act via a distinct mechanism of action from that of currently used antibiotics [8], nanomaterials have been regarded as alternatives to antibiotics that do not give rise to resistant strains [9]. However, certain nanomaterials tend to exhibit toxicity, with prolonged exposure to nanomaterials potentially having an adverse effect on human health [10]. Therefore, further research on the development of nanomaterial-based antibiotics should be conducted to overcome the above-described limitations and avoid the production of detrimental environmental contaminants. For this purpose, researchers have extensively developed novel biomimetic nanoparticles (NPs) coated with various cell-derived membranes for non-hazardous and environmentally friendly applications within the biomedical, environmental, and food industries [11]. In this regard, NPs enveloped with cell membranes not only retain the adjustable physicochemical features of synthetic NPs, but also the bio-interfacial properties and beneficial activities of biological membranes. NP-coated membranes from red blood cells [12], leukocytes [13], platelet [14], cancer cells [15], and bacteria [11] have been investigated for the aforementioned applications.

Among the cellular membranes utilized for cloaking NP-associated platforms, bacteria-derived extracellular vesicles (EVs), including outer membrane vesicles (OMVs) from Gram-negative bacteria or nanovesicles (NVs) from Gram-positive bacteria, are comparatively novel options. OMVs were previously considered to be derived from Gram-negative bacteria only due to their outer membrane layer [16]. However, NVs from Gram-positive bacteria cells, including *S. aureus* [17], *Streptomyces lividans* [18], *Listeria monocytogenes* [19], *Bacillus subtilis* [20], *Lactobacillus plantarum* [21], *L. reuteri* DSM 17938 [22], *L. paracasei* [23], and other species, have also been isolated.

The importance of bacterial membrane-cloaked nanoplatforms has been highlighted in vaccine development as these membranes are enriched in bioactive proteins, toxins, virulence factors, and immunogenic materials, which can stimulate bacteria-host interactions [11]. In addition, EVs with identical membrane structures to that of parental bacteria are non-replicative [24]. Furthermore, the poor cell-penetrating ability of nanomaterials can be readily enhanced by OMVs, since bacterial cells recognize OMVs as their own constituents [11]. In principle, while EV-coated NPs harbor the great potential for the targeted killing of bacterial cells, only a few pathogen-derived EV-coated NPs have been reported to show desired properties for antibacterial agents, which include specificity, low toxicity, and stimuli responsiveness [25,26].

Even though pathogen-derived EVs are regarded as safe following detoxification, their potential effects on human health remain unclear [27,28]. EVs sourced from generally regarded as safe (GRAS)-grade bacteria may overcome these limitations. *Lactobacillus* species are one of the most widely used non-pathogenic GRAS-grade probiotic species [29]. For instance, these bacteria have been used for fermentation in the food production industry for decades due to their ability to grow aerobically and anaerobically, in addition to their lactic acid-fermenting metabolism. The beneficial effects of *Lactobacillus* have fostered their use in various applications, including environmental issues [30,31]. It should be noted that *Lactobacillus* species and their EVs harbor innate antibacterial activity against *S. aureus* [21,32,33]. Therefore, *Lactobacillus*-derived EVs coated with functionalized antibacterial NPs can be a promising platform for developing agents against *S. aureus*.

The potential use of metals (e.g., Ag and Au) and metal oxides (e.g., ZnO and CuO) for antibacterial applications has been extensively reported [34]. In particular, ZnO (ZO) NPs have been utilized for antibacterial activity due to their ability to generate reactive oxygen species (ROS) and disrupt the bacterial cell membrane [35]. However, their toxic nature within nano-range concentrations hinders clinical application in the absence of modifications [36]. In recent years, two-dimensional (2D) layered materials such as graphene [37,38] and black phosphorus [35,39] have emerged as promising antibacterial platforms owing to their distinctive physiochemical properties. Molybdenum disulfide (MoS_2_), another 2D nanomaterial, has exhibited considerable antibacterial activity mediated via physical damage to cells [40]. In addition, coating the surface of NPs with polyethylene glycol (PEG) (PEGylation) and MoS_2_ could improve antibacterial activity and biocompatibility [41]. However, only a few studies have investigated the antibacterial activity of functionally modified MoS_2_ nanosheets to date [40,41].

Inspired by the above-described advantageous characteristics of EVs from *Lactobacillus*, as well as the antibacterial properties of ZnO and functionally modified MoS_2_ nanosheets, we sought to fabricate a PEGylated MoS_2_ nanosheet-ZO NP (MZ) nanocomposite cloaked with *Lactobacillus*-derived EVs and employ it for the selective eradication of *S. aureus* pathogens that threaten human and ecosystem. The MZ nanocomposite was synthesized and fully characterized through X-ray diffraction (XRD), transmission electron microscopy (TEM), and X-ray photoelectron spectroscopy (XPS) analysis. Further, MZ coated with *Lactobacillus*-derived EVs was synthesized, and its antibacterial activity was determined via the 96-well-based microbroth dilution method. Among *Lactobacillus*-derived EVs, only *L. paracasei*-derived EVs coated onto the MZ nanocomposite (MZ-LPEV) stimulated the activity and selectivity of MZ against *S. aureus*, with enhanced ROS generation and membrane disruption when compared to those achieved with other parental NPs and nanocomposites. Furthermore, the MZ-LPEV nanocomposite exhibited greater biocompatibility when compared to that of the ZO NPs and MZ nanocomposites. Taken together, our newly developed MZ-LPEV is the first example that LPEV could be utilized for the preparation of a biocompatible and synergistic nanoantibiotic in killing *S. aureus*.

## 2. Materials and Methods

### 2.1. Synthesis of ZO NPs

ZO NP synthesis was performed as described in a previous report [42] with a low-temperature precipitation process. Initially, an approximate amount of sodium hydroxide (NaOH, 95%, Junsei, Tokyo, Japan) was added dropwise onto the aqueous solution of Zn(NO_3_)_2_·6H_2_O, (98%, Sigma-Aldrich, St. Louis, MO, USA) with continuous stirring. Then, the solution mixture was stirred for 6 h at 80 °C until transferred to an ice bath to stop the reaction. Finally, after washing with DW and ethanol, the precipitate was centrifuged and dried overnight in an air oven at 60 °C.

### 2.2. Synthesis of MZ Nanocomposite

The synthesis of MZ nanocomposite was accomplished through 3 steps: (1) preparation of MoS_2_ nanosheets via aqueous exfoliation of MoS_2_ powder, (2) PEGylation of MoS_2_ nanosheets, and (3) loading of ZO NPs onto the PEG-modified MoS_2_ nanosheets to obtain the functionalized MZ nanocomposite.

1st step: A previously developed method was utilized to prepare the MoS_2_ nanosheets via the ultrasonication-assisted aqueous exfoliation of MoS_2_ powder (<2 mm, 99%; Sigma Aldrich) [43]. Initially, ~100 mg of MoS_2_ was added to 20 mL of 1-methyl-2-pyrrolidinone (NMP, 99%; Sigma-Aldrich, Saint Louis, MO, USA) solvent. Next, ultrasonication (~6 h) was employed to achieve direct exfoliation of MoS_2_ in an ice bath ultrasonicator (60 Hz; UltraSonic Cleaner 100H, Shinhan-Sonic, Incheon, Korea) to prevent overheating. Subsequently, centrifugation (2000 rpm for 10 min, 4 °C) was utilized to remove the unexfoliated MoS_2_, and the supernatant was centrifuged again at 10,000 rpm for 10 min at 4 °C. Finally, the directly exfoliated MoS_2_ nanosheets (3.5 mg·mL^−1^) were stored in DW for further use after discarding the sediment.

2nd step: To prepare PEGylated MoS_2_ nanosheets, 2 mL of MoS_2_ nanosheets (3.5 mg·mL^−1^) was mixed with 100 mg of PEG 8000 (Sigma-Aldrich, Saint Louis, MO, USA) in 30 mL DW under continuous stirring for ~4 h at 25 °C. Thereafter, the PEG-modified MoS_2_ nanosheets were collected via centrifugation and directly used in the next step.

3rd step: The collected PEGylated MoS_2_ nanosheets were redispersed in 30 mL DW. Then, 100 mg of synthesized ZO NPs was also dispersed in the same reaction mixture and continuously stirred overnight. Finally, the MZ nanocomposite was collected via centrifugation and dried inside a vacuum air oven at 60 °C for 4 h.

### 2.3. Material Properties

XRD was employed to assess the diffraction patterns of ZO NPs and the MZ nanocomposite. An X-ray diffractometer (D8 Advance with DAVINCI design X-ray diffraction unit, Bruker, Berlin, Germany) with a nickel-filtered Cu Kα radiation source (λ = 1.5406 Å) in the 2θ range of 5–80° was utilized for the same. In addition, microstructural analysis of the representative sample MZ was performed via TEM (Bruker Nano GmbH, Berlin, Germany) using carbon-coated 300-mesh Cu grids. The AXIS Supra XPS microprobe surface analysis system was utilized to assess the MZ nanocomposite. The binding energy range of 200–1200 eV was used for the scanning to determine the chemical state of elements. The C 1s peak position at 284.5 eV was used as the binding energy reference.

### 2.4. Bacterial Strains

*Acinetobacter baumannii* (ATCC 19606), *Escherichia coli* (ATCC 25922), *L. paracasei* subsp. *tolerans* (ATCC 25599), *Pseudomonas aeruginosa* (ATCC 27853), *S. aureus* (ATCC 25923), *S. epidermidis* (ATCC 12228), and *S. saprophyticus* (ATCC 15305) were purchased from American Type Culture Collection (ATCC, Manassas, VA, USA; www.atcc.org, accessed on 23 October 2022). *L. acidophilus* (KCTC 3164), *L. fructosus* (KCTC 3544), and *L. plantarum* (KCTC 3107) were purchased from Korean Collection for Type Cultures (KCTC, Jeong-eup, Korea; https://kctc.kribb.re.kr, accessed on 23 October 2022).

### 2.5. Preparation of Bacteria-Derived EVs

*L. acidophilus* (KCTC 3164), *L. fructosus* (KCTC 3544), *L. plantarum* (KCTC 3107), and *L. paracasei* subsp. *tolerans* (ATCC 25599) cells were cultured in BD Difco^TM^ *Lactobacilli* MRS broth (Thermo Fisher Scientific, Waltham, MA, USA) at 37 °C with shaking at 230 rpm to an optical density at 600 nm (OD_600_) of 1.5. The bacterial cultures were pelleted at 4000× *g* for 30 min at 4 °C. The cell-free supernatants were collected and filtered using a 0.22 μm Syringe Filter (Biofact, Daejeon, Korea). EVs from the supernatants were isolated using the ExoBacteria^TM^ OMV Isolation Kit (System Biosciences, Palo Alto, CA, USA) according to the manufacturer’s instructions, and the final product was resuspended in PBS buffer. EVs were stored at 4 °C until use.

### 2.6. Evaluation of Antibacterial Activity

The minimum inhibitory concentration (MIC) of samples was determined *in vitro* using a 96-well plate format microbroth dilution method as previously described [35,44]. Bacterial cells of various concentrations were grown in Mueller–Hinton Broth (MHB) at 37 °C for 16 h with shaking for each MIC measurement. To evaluate the antibacterial activity of GRAS-grade bacteria-derived EVs against *S. aureus*, a standard zone of inhibition (ZOI) assay was performed as described in a previous report [45]. Here, 20 µL (10 mg·mL^−1^) EVs and Mueller–Hinton Broth (MHB) agar plates inoculated with bacterial cell suspensions (0.5 McFarland turbidity) were used. The bactericidal activity of materials was characterized by spotting aliquots of sample-treated bacterial cultures on Luria Bertani (LB)-agar plates [39]. Checkerboard assays were employed to verify the synergistic action of materials by determining the fractional inhibitory concentration index/indices (FICI) [35,44]. A representative from triplicate experiments is shown.

### 2.7. Preparation of MZ-Lactobacillus-Derived EVs

The same volumes (100 µL) of MZ nanocomposite and EVs at a concentration of 2 mg·mL^−1^ were mixed in a microcentrifuge tube to adjust the MZ nanocomposite concentration at 1 mg·mL^−1^. In order to obtain MZ-*Lactobacillus*-derived EVs, the dispersion was sonicated in a water-bath ultrasonicator for 3 min before further use for evaluating antibacterial activity. Coating was performed as described in previous work [25]. MZ-*Lactobacillus*-derived EVs were named MZ-LPEV: *L. paracasei*-derived extracellular vesicle-cloaked MZ; MZ-LMEV: *L. plantarum*-derived extracellular vesicle-cloaked MZ; MZ-LFEV: *L. fructosus*-derived extracellular vesicle-cloaked MZ; MZ-LAEV: *L. acidophilus*-derived extracellular vesicle-cloaked MZ.

### 2.8. Characterization of LPEV and MZ-LPEV

The size (diameter) of MZ, LPEV, and MZ-LPEV was determined via nanoparticle tracking analysis (NTA) using NanoSight NS300 (Malvern Panalytical, Malvern, UK) with a 532 nm light source according to manufacturer’s instructions [46]. The physical properties of samples as zeta potential (millivolts, mV) were analyzed using a Nanopartica SZ-100 (Horiba Ltd., Kyoto, Japan) according to the manufacturer’s instructions. The electrophoretic mobility μ at 25 °C in 1 mM NaCl was determined using Smoluchowski’s equation ζ = μη/ε, where η is the medium viscosity and ε the medium dielectric constant. Zeta potential values shown for individual samples are the means of at least three measurements with standard deviation (*p* < 0.05).

### 2.9. Morphological Characterization of Bacteria

The morphological changes of *S. aureus* bacterial cells [35] were examined after treatment with MZ, LPEV, and MZ-LPEV samples. For this purpose, all nanomaterials at a fixed concentration (sublethal MIC) were added to the bacterial cell suspensions, as described in Section 2.6. Thereafter, the bacterial cells were incubated at 37 °C for 16 h. The resulting cultures were collected via centrifugation at 12,000 rpm for 1 min, followed by resuspension in 500 µL of phosphate-buffered saline (PBS), pH 7, containing 2% formaldehyde and 1% glutaraldehyde. Cell morphology was fixed by incubating samples for 5 min at room temperature. The cell pellets were obtained via centrifugation, washed twice with DW, and resuspended in 1 mL of DW. A 5 µL aliquot was collected from the suspension, deposited on a silicon wafer (5 × 5 mm, Namkang Hi-Tech Co., Ltd., Seongnam, Korea), and allowed to dry at room temperature. VEGA3, a versatile tungsten thermionic emission scanning electron microscopy (SEM) system (TESCAN, Fuveau, France), was used to analyze the dried wafer according to the manufacturer’s protocol.

### 2.10. Measurement of ROS Production

ROS production capacity of the MZ-LPEV nanocomposite against *S. aureus* was evaluated based on the previous report [39]. Initially, bacterial cells of 0.5 McFarland turbidity in PBS were treated with MZ, LPEV, and MZ-LPEV samples at 2.5 µg·mL^−1^ in the presence of 2′,7′-dichlorodihydrofluorescin diacetate (DCFH-DA) (Sigma-Aldrich, Burlington, MA, USA) at a final concentration of 30 µM in PBS. Next, the 96-well plate containing bacterial cell cultures was incubated at 37 °C for 2 h with vigorous shaking (500 rpm). The amount of ROS was determined based on fluorescence intensity with excitation and emission wavelengths of 485 and 520 nm, respectively, using FLUOstar Omega (BMG Labtech, Ortenberg, Germany). A bacterial cell suspension in PBS without treatment was used as a control. MARS Data Analysis software (ver. 3.02 R2, BMG Labtech, Ortenberg, Germany) was used to further analyze the samples. The measurements were taken in triplicate, and the relative ROS production of treated samples was compared to the control; the averaged values with standard deviation (*p* < 0.05) are shown.

### 2.11. In Vitro Cytotoxicity Assay

The cytotoxicity of ZO NP, MZ, and MZ-LPEV was evaluated via the colorimetric WST-1 Cell Proliferation assay using a WST assay kit (Ez-Cytox; Dogenbio, Seoul, Korea), according to the vendor’s protocol [35]. Human embryonic kidney (HEK 293) cells were used, purchased from the ATCC (Manassas, VA, USA; www.atcc.org, accessed on 23 October 2022) and maintained in RPMI1640 medium (Thermo Fisher Scientific, Waltham, MA, USA) with 10% of fetal bovine serum (Thermo Fisher Scientific, Waltham, MA, USA) at 37 °C and 5% CO_2_. Cells were seeded into 96-well plates at a density of 5000 cells per well and incubated for 24 h at 37 °C. Thereafter, cells were further incubated for 48 h in the presence of MZ and MZ-LPEV samples at the concentration of 10 and 25 µg·mL^−1^ (4–10 times MIC of MZ-LPEV) in 0.1% dimethyl sulfoxide. Subsequently, the cells were incubated with WST-1 reagent (one-tenth of the medium volume). A spectrophotometric microplate reader (BMG LABTECH GmbH, Ortenber, Germany) was then used to determine the amount of formazan dye formed by measuring the absorbance at 450 nm. The mean values of triplicate measurements with standard deviation (*p* < 0.05) are shown.

## 3. Results and Discussion

### 3.1. Material Properties

#### 3.1.1. Phase Structure

**Figure 1:**
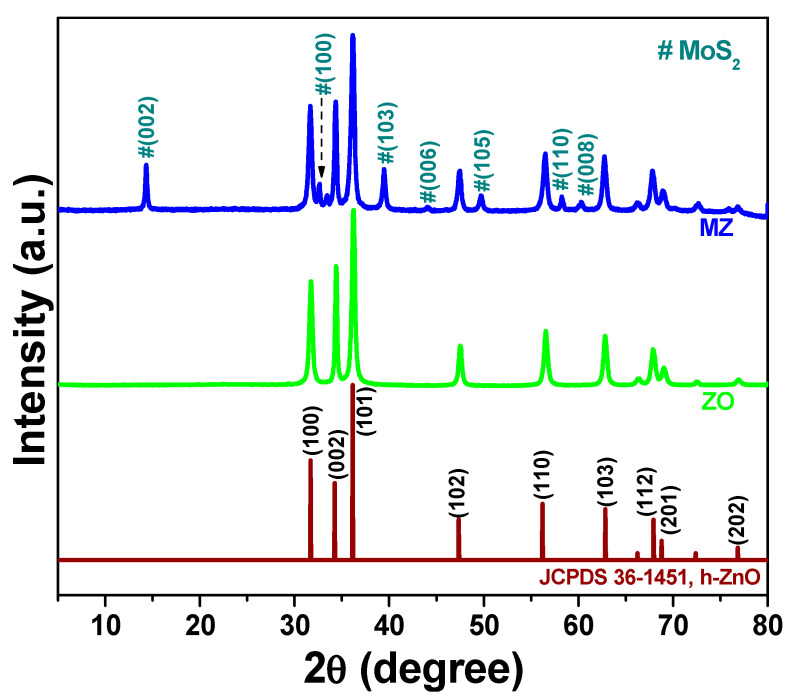
X-ray diffraction (XRD) patterns of ZO and MZ samples.

Figure 1 shows the crystalline structure of synthesized ZnO (ZO) nanoparticles (NPs) and functionalized MZ nanocomposites, as determined via XRD characterization. The XRD pattern of the ZO sample shows diffraction peaks, which correspond well with hexagonal ZnO [JCPDS 36-1451] [38]. The ZO NPs maintained fine crystalline structures in the MZ nanocomposite. Moreover, some additional diffraction peaks were also visible in the MZ sample, corresponding to the (002), (100), (103), (006), (105), (110), (008) crystal planes of few-layer MoS_2_ nanosheets (ICDD card No.37-1492) [47] and marked with ‘#’ marker to distinguish itself from the peaks of ZO NPs. Taken together, the successful formation of the MZ nanocomposite was confirmed based on XRD characterization results.

#### 3.1.2. Morphology and Microstructure

**Figure 2:**
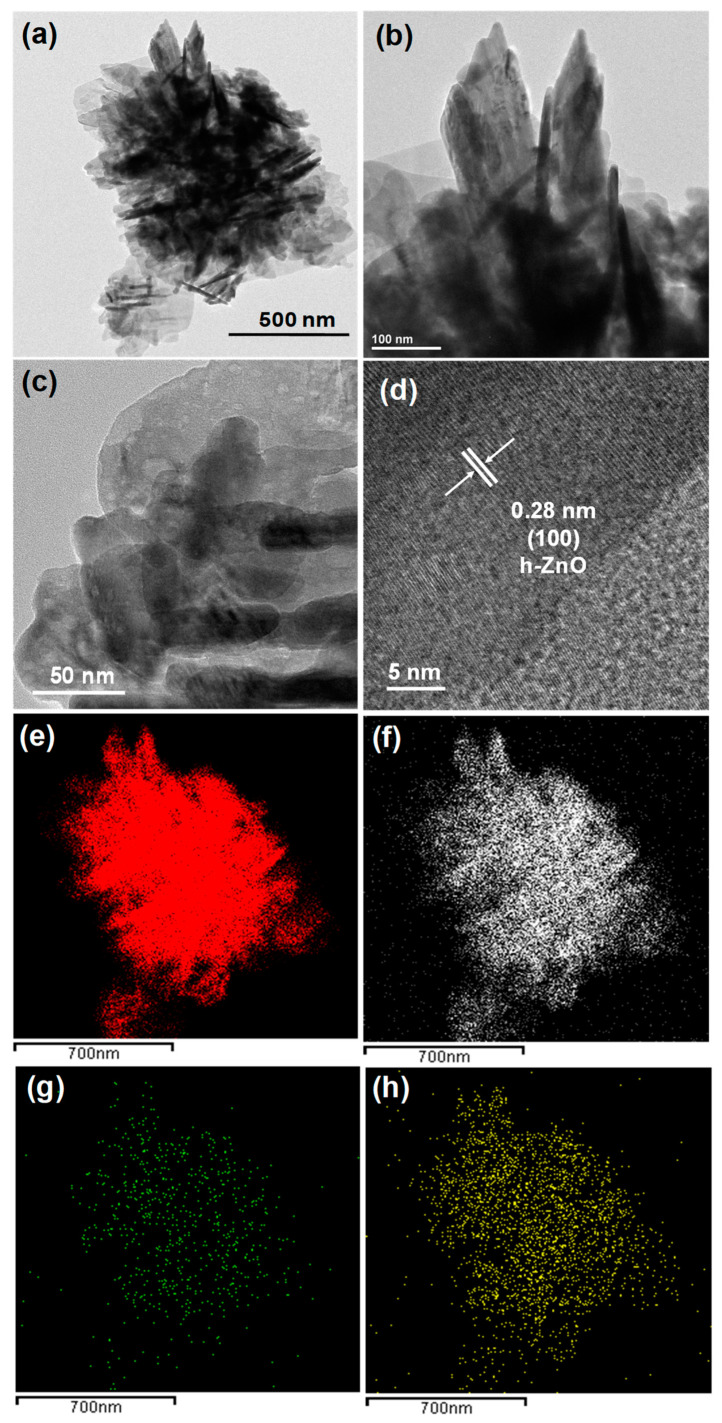
Transmission electron microscopy (TEM) images (**a**–**c**), (**d**) corresponding high-resolution TEM image, and elemental mapping of (**e**) Zn, (**f**) O, (**g**) Mo, and (**h**) S for the MZ nanocomposite.

The morphology of the synthesized MZ nanocomposite was characterized via TEM and is illustrated in Figure 2. Figure 2a–c depicts TEM images of the MZ nanocomposite, where the ZO NPs are clearly distributed on the MoS_2_ nanosheets. The existence of distinct lattice fringes with an interplanar distance of 0.28 nm in the high-resolution TEM (HRTEM; Figure 2d) image of the representative MZ nanocomposite can correspond to the (100) plane of ZnO [42], confirming the presence of ZO NPs in the nanocomposite. Elemental mapping images of the MZ nanocomposite are also depicted in the figure and showed a good distribution of Zn (Figure 2e), O (Figure 2f), Mo (Figure 2g), and S (Figure 2h). Hence, the successful formation of the MZ nanocomposite was confirmed based on TEM and HRTEM results, which corroborated those obtained via XRD.

#### 3.1.3. XPS Results

**Figure 3:**
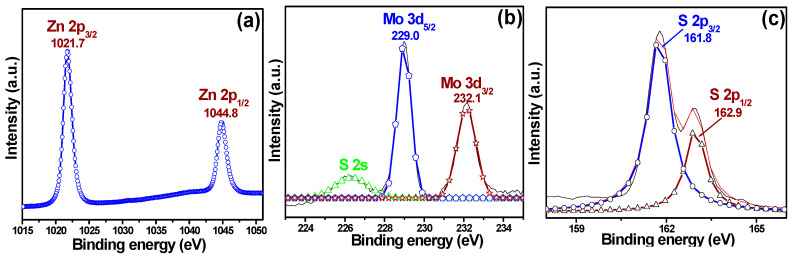
X-ray photoelectron spectroscopy (XPS) results for the MZ nanocomposite: (**a**) Zn 2p spectrum and Gaussian-fitted (**b**) Mo 3d and (**c**) S 2p spectra.

XPS was employed to confirm the chemical composition of the MZ nanocomposite as well as the valence states of elements present in the sample; results are shown in Figure 3. Figure 3a depicts the Zn 2p spectrum, where two strong peaks at binding energies of 1021.7 and 1044.8 eV are visible and can be assigned to Zn 2p_3/2_ and Zn 2p_1/2_, respectively [37]. In addition, the energy difference between the Zn 2p_3/2_ and Zn 2p_1/2_ binding energies is ~23.1 eV, proving the presence of a Zn^2+^ valence state [37] in the MZ nanocomposite. Results presented in Figure 3b, which shows the Gaussian fitting of the Mo 3d spectrum, reveal two characteristic peaks at 229.0 and 232.1 eV. These can be assigned to Mo 3d_5/2_ and Mo 3d_3/2_, respectively [48]. Besides, an additional peak at 226.5 eV was also observed, which corresponds to the S 2s. Likewise, two peaks at 161.8 and 162.9 eV in the S 2p spectrum can also be seen and assigned to S 2p_3/2_ and S 2p_1/2_, respectively [48]. Therefore, the XPS result confirmed the successful formation of the MZ nanocomposite and was in agreement with the XRD result (Figure 1) and TEM (Figure 2) data.

### 3.2. Characterization of MZ-LPEV

To characterize the particle size change of the MZ-LPEV sample after coating, we employed NTA, a method for visualizing and analyzing particles in liquids that relates the rate of Brownian motion to particle size using a microscope camera [46]. We subjected LPEV and MZ-LPEV to NTA while comparing them with each other. The mean values obtained by comparing sizes from D90 samples (90% of the total volume of material in the sample is contained) [46,49] of triplicate capturing were 160.4 and 465.2 nm for LPEV, and MZ-LPEV, respectively (Table 1). This indicated that the size of MZ-LPEV was much bigger than that of LPEV, confirming the coating of MZ with LPEV. These findings highlighted the importance of LPEV coating onto the MZ nanocomposite, which not only stabilized the nanocomposite but also potentiated its antibacterial activity (discussed in the latter section). In separate experiments, zeta potential measurements (Table 1) were performed to characterize the physical difference between MZ and MZ-LPEV. The zeta potential values of MZ, LPEV, and MZ-LPEV were −43.7, −19.9, and −21.5 mV, respectively. This indicated a similarity between LPEV with MZ-LPEV in terms of zeta potential, in addition to confirming the coating of MZ with LPEV. Overall, NTA and zeta potential analyses proved the formation of the LPEV-coated MZ nanocomposite (MZ-LPEV).

### 3.3. Evaluation of Anti-S. aureus Activity of MZ-Lactobacillus-Derived EVs

*Lactobacillus* species exhibit anti-*S. aureus* activity [32,33]. In addition, EVs derived from *Lactobacillus* species could be used as natural anti-bacterial materials [21]. However, we could not find any clear ZOI for *Lactobacillus*-derived EVs against the *S. aureus* ATCC 25923 strain. This suggested that *Lactobacillus*-derived EVs could be used to achieve a synergistic effect with MZ against *S. aureus*.

To verify this notion, MZ-*Lactobacillus* EV nanocomposites (MZ-LPEV, MZ-LMEV, MZ-LFEV, and MZ-LAEV) were prepared, and their MIC values against *S. aureus* were determined (Table 2). Subsequently, the bactericidal activity of MZ, LPEV, and MZ-LPEV from corresponding MIC plates was assessed. MZ and MZ-LPEV exhibited MICs of 10 and 2.5 μg·mL^−1^, respectively; however, LPEV itself did not exhibit antibacterial efficacy against *S. aureus* up to 100 μg·mL^−1^ (Figure S1). Compared to the MIC of ZO NPs against *S. aureus* (50 μg·mL^−1^) (Table 2; Figure S2), the MIC of the MZ nanocomposite was 10 μg·mL^−1^ (Figures S1 and S2), indicating that functionalization of ZO NPs via PEGylated MoS_2_ enhanced their bactericidal activity against *S. aureus* 5-fold. Next, the MICs of MZ-*Lactobacillus*-derived EV nanocomposites against *S. aureus* were determined. Only MZ cloaked with LPEV (MZ-LPEV) enhanced the anti-*S. aureus* activity of MZ 4-fold, thereby decreasing the MIC to 2.5 μg·mL^−1^ (Table 2; Figures S1 and S2). The MIC of MZ-LAEV and MZ-LMEV was 100 μg·mL^−1^, while that of MZ-LFEV was 50 μg·mL^−1^, antagonizing the MZ activity ~5–10 fold (Table 2; Figure S2). The result itself shows that the ZO NP, with MIC of 50 μg·mL^−1^, is an effective antibacterial agent against *S. aureus*. Additionally, the MoS_2_ and LPEV together potentiated ZO NPs activity against *S. aureus* 20-fold, with LPEV promoting antibacterial specificity. The excellent antibacterial activity and high specificity of the LPEV-coated MZ nanocomposite were attributed to the enhanced uptake of the MZ-LPEV nanocomposite by *S. aureus* cells. However, it is also possible that the mixture of MZ with LPEV, rather than the MZ-LPEV single nanocomposite, could exhibit synergistic activity against *S. aureus*. To verify this notion, checkerboard assays with LPEV and MZ nanocomposite were performed, thus revealing no synergy between LPEV and MZ against *S. aureus* without any change in the MIC of MZ (10 μg·mL^−1^) (Figure S3). Overall, our results show that the LPEV coating of MZ as a combined nanocomposite conferred potent and selective synergistic activity to the inherent antibacterial properties of ZO and MoS_2_.

### 3.4. Species Selectivity of MZ-LPEV

The selective antibacterial activity of MZ, LPEV, and MZ-LPEV nanocomposites was evaluated by determining MICs against different standard Gram-positive strains dwelling with *S. aureus* (*S. epidermidis* and *S. saprophyticus*; Figure S4) as well as Gram-negative bacterial strains (*A. baumannii*, *E. coli*, and *P. aeruginosa*; Figure S5). As shown in Figures S4 and S5, MZ-LPEV was not synergistically active against the bacterial strains except *S. aureus* (ATCC 25923). This indicated that MZ-LPEV is a potential and selective antibacterial agent against *S. aureus*. More interestingly, the MIC of MZ-LPEV against *S. aureus* was 4-fold higher compared to that of other MZ nanocomposites with EVs derived from other *Lactobacillus* strains (Table 2), indicating that LPEV potentiates both the activity and selectivity of the MZ nanocomposite against *S. aureus*. Taken together, MZ-LPEV could have the potential as a nanoplatform against *S. aureus*.

### 3.5. Plausible Antibacterial Mechanism

**Figure 4:**
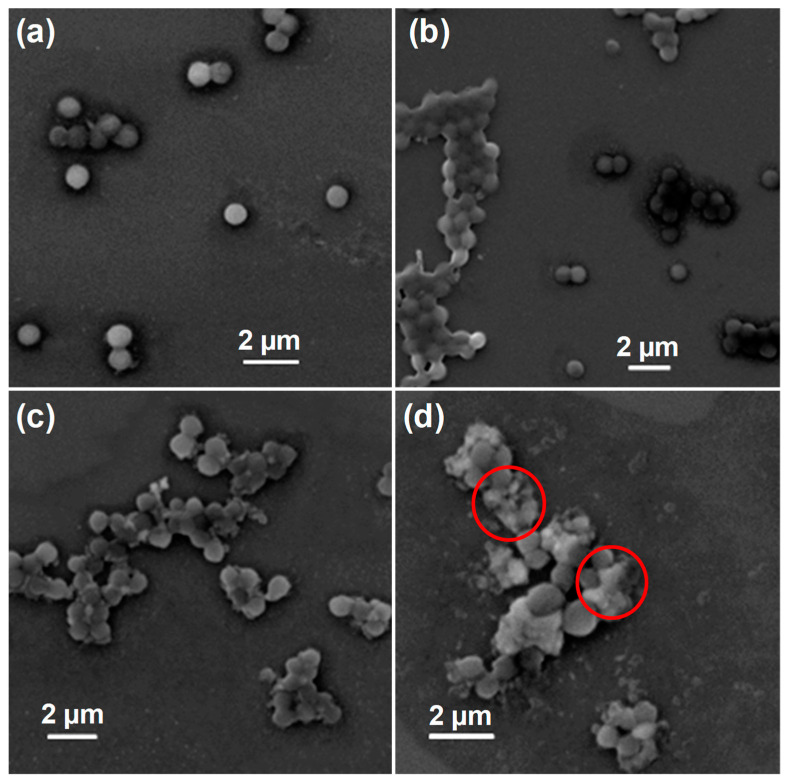
Scanning electron microscopy images of *S. aureus* after various treatments. (**a**) Untreated, (**b**) LPEV-treated, (**c**) MZ-treated, and (**d**) MZ-LPEV-treated. The red circles indicate morphological changes in bacterial cells.

**Figure 5:**
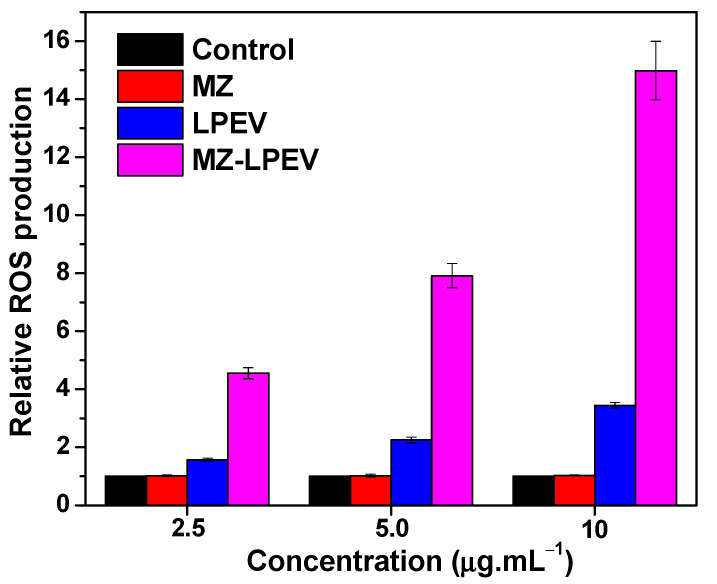
Relative ROS production. Fluorescence intensities at 520 nm were measured for *S. aureus* (ATCC 25923) cells treated with MZ, LPEV, and MZ-LPEV, respectively, and a relative ratio to the control sample is shown. Bacterial cell suspension in PBS without treatment was used as a control. MARS Data Analysis software (ver. 3.02 R2; BMG Labtech GmbH, Ortenber, Germany) was used for data processing and averaged values of relative ROS production from triplicate experiments are shown (*p* < 0.05).

Disruption of the bacterial cell membrane and ROS generation are well-known and widely established antibacterial mechanisms of nanomaterials [35,39]. Therefore, the morphological changes (Figure 4) of bacterial cells (*S. aureus*) treated with MZ, LPEV, and MZ-LPEV were evaluated to confirm the antibacterial mechanism of action at play. The ROS production ability (Figure 5) of samples was also assessed to further verify the antibacterial mechanism of MZ-LPEV.

#### 3.5.1. Morphological Characterization of Bacteria

SEM was utilized to assess the morphological changes of *S. aureus* (Figure 4) under treatment with nanomaterials and LPEV. As expected, the untreated *S. aureus* (Figure 4a) bacterial cells displayed a smooth surface without disruption. When bacterial cells were treated with LPEV, the bacterial cell surface was smooth as in the control condition; a population of grouped cells was also detected (Figure 4b), suggesting that the LPEV itself does not exert antibacterial effects and might promote attachment between cells. However, bacterial cells under treatment with the MZ nanocomposite exhibited membrane disruption, as shown in Figure 4c. The phenotype was more pronounced under treatment with MZ-LPEV (Figure 4d). The observed cell membrane disruption can be attributed to ZO NPs [35], the MoS_2_ nanosheet [40], and LPEV attaching to the bacterial cell surface. Hence, this result confirmed that the synergistic antibacterial activity of the MZ-LPEV nanocomposite against *S. aureus* is mediated via bacterial membrane disruption.

#### 3.5.2. ROS Production

ROS production via nanomaterials damages various intracellular components of bacteria (DNA, proteins, or other functional cellular components), resulting in cell death [50]. To determine whether MZ-LPEV increases ROS production against *S. aureus*, ROS production of the MZ-LPEV nanocomposite was measured at a fluorescence intensity of 520 nm and then compared to that of the MZ nanocomposite and LPEV at multiple concentrations (Figure 5). As shown in Figure 5, ROS production via the MZ nanocomposite against *S. aureus* cells did not change at all tested concentrations. Thus, the MZ nanocomposite itself had no ROS production capacity. Meanwhile, ROS production by LPEV was 1.6 to 3.4 times higher than the control and showed a concentration-dependent increase. More interestingly, ROS production by the MZ-LPEV nanocomposite at 2.5 μg·mL^−1^ (MIC) was 4.5 times greater than that of the control sample and similar to that achieved by LPEV at 10 μg·mL^−1^. ROS production by MZ-LPEV was concentration-dependent and ~3 times greater at 10 μg·mL^−1^ when compared to the MIC. This higher ROS production from MZ-LPEV can be attributed to the formation of nanocomposite and the synergistic capability of nanocomposite in terms of antibacterial properties [51,52]. This indicated that MZ nanocomposite coated with LPEV (MZ-LPEV) exhibited strong synergistic activity when compared to individual samples. The 4-fold increase of synergistic antibacterial activity observed for the MZ-LPEV nanocomposite (Table 1) relative to individual mixtures (Figure S3) could therefore be attributed to enhanced ROS production.

### 3.6. In Vitro Cytotoxicity of Materials

**Figure 6:**
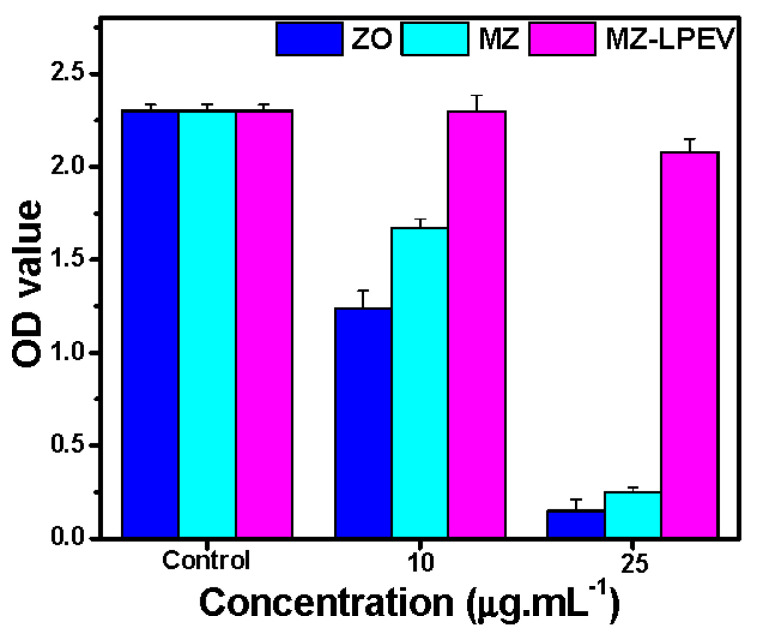
Viability (WST assay) of the human embryonic kidney (HEK 293) cells treated with ZO, MZ, and MZ-LPEV at various concentrations for 48 h. Averaged values from triplicate experiments are shown (*p* < 0.05).

One of the key criteria for nanocomposite application is biocompatibility. To evaluate biocompatibility, WST-1 assays (Figure 6) were performed to determine the cytotoxic effects of MZ and MZ-LPEV nanocomposite at varying concentrations on HEK293 cells, for which the penetration ability and cellular interactions of ZO NPs have been well defined [53]. As shown in Figure 6, the cell viability under MZ treatment was ~25% greater when compared to that under ZO NPs at 10 µg·mL^−1^, which is 4-times the MIC (2.5 µg·mL^−1^) and thus indicative of greater biocompatibility. However, the feature was not retained at 25 µg·mL^−1^, and >90% of cells were non-viable. Meanwhile, cells treated with MZ-LPEV remained viable at 10 µg·mL^−1^ and even 25 µg·mL^−1^. Therefore, LPEV, as part of the MZ-LPEV nanocomposite, helps alleviate the toxicity of ZO NPs and the MZ nanocomposite. Taken together, our newly developed MZ-LPEV nanocomposite is a potent and biocompatible antibacterial nanoplatform against *S. aureus*.

## 4. Conclusions

In summary, we successfully prepared a nanocomposite comprising PEG-modified-MoS_2_/ZO NPs cloaked with *L. paracasei*-derived EVs (MZ-LPEV), which we then characterized as a selective and biocompatible antibacterial platform against hazardous *S. aureus* species. This nanocomposite exhibited superb synergistic activity by utilizing the advantages of both the functionalized MZ and LPEV, which resulted in enhanced ROS production and membrane disruption. Overall, our study demonstrated the specific usage of *L. paracasei*-derived EVs for selective targeting by the modified 2D antibacterial nanocomposite. Therefore, LPEV clocked with multiple 2D antibacterial nanocomposites represents a unique platform for the nanoantibiotic remediation of *S. aureus* infection and transmission across environmental matrices. Since different types of 2D nanocomposites would have distinct selectivity, our new strategy holds promise for the treatment of different bacterial infections that are harmful to the environment.

## Figures and Tables

**Table 1:** Characterization of MZ, LPEV, and MZ-LPEV. Hydrodynamic size (Mean diameter, nm) from NTA analysis and zeta potential values were shown. Data are presented as mean ± standard deviation (*p* < 0.05).

Sample	Size (nm)	Zeta Potential (mV)
MZ	Not measured	−43.7 ± 0.4
LPEV	160.4 ± 13.1	−19.93 ± 0.35
MZ-LPEV	465.2 ± 12.4	−21.5 ± 2.8

**Table 2:** MIC values of ZO NP, MZ, and *Lactobacillus* strain EV-coated MZ against *S. aureus* (ATCC 25923) ^1^.

Sample	MIC (µg·mL^−1^)
ZO NP	50
MZ	10
MZ-LPEV	2.5
MZ-LMEV	100
MZ-LFEV	50
MZ-LAEV	100

^1^ Data shown here are representative of the triplicate experiments. Abbreviations: MIC, Minimum inhibitory concentration; ZO, Zinc Oxide; NP, Nanoparticle; MZ, PEGylated MoS_2_-ZO NP; LPEV, *Lactobacillus paracasei*-derived extracellular vesicle; MZ-LPEV, LPEV-cloaked MZ; LMEV, *Lactobacillus plantarum*-derived extracellular vesicle; MZ-LMEV, LMEV-cloaked MZ; LFEV, *Lactobacillus fructosus*-derived extracellular vesicle; MZ-LFEV, LFEV-cloaked MZ; LAEV, *Lactobacillus acidophilus*-derived extracellular vesicle; MZ-LAEV, LAEV-cloaked MZ.

## Data Availability

Not applicable.

## Supplementary Materials

The following supporting information can be downloaded at: https://www.mdpi.com/article/10.3390/pharmaceutics14112273/s1, Figure S1: Bactericidal activity of nanocomposites; Figure S2: Antibacterial activity of nanomaterials and LPEV; Figure S3: Evaluation of synergistic activity between MZ and LPEV; Figure S4: Antibacterial activity of nanocomposites and MZ-LPEV against Gram-positive bacteria; Figure S5: Antibacterial activity of nanocomposites and MZ-LPEV against Gram-negative bacteria.

## References

[1] Kavanaugh J.S., Horswill A.R. (2016). Impact of environmental cues on *Staphylococcal* quorum sensing and biofilm development. J. Biol. Chem..

[2] Denissen J., Reyneke B., Waso-Reyneke M., Havenga B., Barnard T., Khan S., Khan W. (2022). Prevalence of ESKAPE pathogens in the environment: Antibiotic resistance status, community-acquired infection and risk to human health. Int. J. Hyg. Environ. Health.

[3] Kozajda A., Jezak K., Kapsa A. (2019). Airborne *Staphylococcus aureus* in different environments-a review. Environ. Sci. Pollut. Res..

[4] Wertheim H.F., Melles D.C., Vos M.C., van Leeuwen W., van Belkum A., Verbrugh H.A., Nouwen J.L. (2005). The role of nasal carriage in *Staphylococcus aureus* infections. Lancet Infect. Dis..

[5] Zhu F., Zhuang H., Ji S., Xu E., Di L., Wang Z., Jiang S., Wang H., Sun L., Shen P. (2021). Household transmission of community-associated methicillin-resistant *Staphylococcus aureus*. Front. Public Health.

[6] Lee A.S., de Lencastre H., Garau J., Kluytmans J., Malhotra-Kumar S., Peschel A., Harbarth S. (2018). Methicillin-resistant *Staphylococcus aureus*. Nat. Rev. Dis. Primers.

[7] DeLeo F.R., Chambers H.F. (2009). Reemergence of antibiotic-resistant *Staphylococcus aureus* in the genomics era. J. Clin. Investig..

[8] Matai I., Sachdev A., Dubey P., Kumar S.U., Bhushan B., Gopinath P. (2014). Antibacterial activity and mechanism of Ag-ZnO nanocomposite on *S. aureus* and GFP-expressing antibiotic resistant *E. coli*. Colloids Surf. B Biointerfaces.

[9] Naskar A., Kim K.-S. (2019). Nanomaterials as delivery vehicles and components of new strategies to combat bacterial infections: Advantages and limitations. Microorganisms.

[10] Ganguly P., Breen A., Pillai S.C. (2018). Toxicity of nanomaterials: Exposure, pathways, assessment, and recent advances. ACS Biomater. Sci. Eng..

[11] Naskar A., Cho H., Lee S., Kim K.-S. (2021). Biomimetic nanoparticles coated with bacterial outer membrane vesicles as a new-generation platform for biomedical applications. Pharmaceutics.

[12] Malhotra S., Dumoga S., Singh N. (2022). Red blood cells membrane-derived nanoparticles: Applications and key challenges in their clinical translation. Wiley Interdiscip. Rev. Nanomed..

[13] Wang D., Wang S., Zhou Z., Bai D., Zhang Q., Ai X., Gao W., Zhang L. (2022). White blood cell membrane-coated nanoparticles: Recent development and medical applications. Adv. Healthc. Mater..

[14] Han H., Bártolo R., Li J., Shahbazi M.A., Santos H.A. (2022). Biomimetic platelet membrane-coated nanoparticles for targeted therapy. Eur. J. Pharm. Biopharm..

[15] Harris J.C., Scully M.A., Day E.S. (2019). Cancer cell membrane-coated nanoparticles for cancer management. Cancers.

[16] Guerrero-Mandujano A., Hernández-Cortez C., Ibarra J.A., Castro-Escarpulli G. (2017). The outer membrane vesicles: Secretion system type zero. Traffic.

[17] Wang X., Koffi P.F., English O.F., Lee J.C. (2021). *Staphylococcus aureus* extracellular vesicles: A story of toxicity and the stress of 2020. Toxins.

[18] Schrempf H., Merling P. (2015). Extracellular *Streptomyces lividans* vesicles: Composition, biogenesis and antimicrobial activity. Microb. Biotechnol..

[19] Woo J.H., Kim S., Lee T., Lee J.C., Shin J.H. (2021). Production of membrane vesicles in *Listeria monocytogenes* cultured with or without sub-inhibitory concentrations of antibiotics and their innate immune responses *in vitro*. Genes.

[20] Brown L., Kessler A., Cabezas-Sanchez P., Luque-Garcia J.L., Casadevall A. (2014). Extracellular vesicles produced by the Gram-positive bacterium *Bacillus subtilis* are disrupted by the lipopeptide surfactin. Mol. Microbiol..

[21] Lee B.H., Wu S.C., Shen T.L., Hsu Y.Y., Chen C.H., Hsu W.H. (2021). The applications of *Lactobacillus plantarum*-derived extracellular vesicles as a novel natural antibacterial agent for improving quality and safety in tuna fish. Food Chem..

[22] Grande R., Celia C., Mincione G., Stringaro A., Di Marzio L., Colone M., Di Marcantonio M.C., Savino L., Puca V., Santoliquido R. (2017). Detection and physicochemical characterization of membrane vesicles (MVs) of *Lactobacillus reuteri* DSM 17938. Front. Microbiol..

[23] Shi Y., Meng L., Zhang C., Zhang F., Fang Y. (2021). Extracellular vesicles of *Lacticaseibacillus paracasei* PC-H1 induce colorectal cancer cells apoptosis via PDK1/AKT/Bcl-2 signaling pathway. Microbiol. Res..

[24] Elsharkasy O.M., Nordin J.Z., Hagey D.W., de Jong O.G., Schiffelers R.M., Andaloussi S.E., Vader P. (2020). Extracellular vesicles as drug delivery systems: Why and how?. Adv. Drug Deliv. Rev..

[25] Gao F., Xu L., Yang B., Fan F., Yang L. (2019). Kill the real with the fake: Eliminate intracellular *Staphylococcus aureus* using nanoparticle coated with its extracellular vesicle membrane as active-targeting drug carrier. ACS Infect. Dis..

[26] Wu S., Huang Y., Yan J., Li Y., Wang J., Yang Y.Y., Yuan P., Ding X. (2021). Bacterial outer membrane-coated mesoporous silica nanoparticles for targeted delivery of antibiotic rifampicin against gram-negative bacterial infection in vivo. Adv. Funct. Mater..

[27] Ortiz A. (2017). Not all extracellular vesicles were created equal: Clinical implications. Ann Transl. Med..

[28] Qin Y., Long L., Huang Q. (2020). Extracellular vesicles in toxicological studies: Key roles in communication between environmental stress and adverse outcomes. J. Appl. Toxicol..

[29] Dean S.N., Leary D.H., Sullivan C.J., Oh E., Walper S.A. (2019). Isolation and characterization of *Lactobacillus*-derived membrane vesicles. Sci. Rep..

[30] Bhogoju S., Nahashon S. (2022). Recent advances in probiotic application in animal health and nutrition: A review. Agriculture.

[31] Di Cerbo A., Palmieri B., Aponte M., Morales-Medina J.C., Iannitti T. (2016). Mechanisms and therapeutic effectiveness of *lactobacilli*. J. Clin. Pathol..

[32] Kang M.S., Lim H.S., Oh J.S., Lim Y.J., Wuertz-Kozak K., Harro J.M., Shirtliff M.E., Achermann Y. (2017). Antimicrobial activity of *Lactobacillus salivarius* and *Lactobacillus fermentum* against *Staphylococcus aureus*. Pathog. Dis..

[33] Shaaban M., Abd El-Rahman O.A., Al-Qaidi B., Ashour H.M. (2020). Antimicrobial and antibiofilm activities of probiotic *Lactobacilli* on antibiotic-resistant *Proteus mirabilis*. Microorganisms.

[34] Guerrero Correa M., Martínez F.B., Vidal C.P., Streitt C., Escrig J., de Dicastillo C.L. (2020). Antimicrobial metal-based nanoparticles: A review on their synthesis, types and antimicrobial action. Beilstein J. Nanotechnol..

[35] Cho H., Naskar A., Lee S., Kim S., Kim K.-S. (2021). A new surface charge neutralizing nano-adjuvant to potentiate polymyxins in killing Mcr-1 mediated drug-resistant *Escherichia coli*. Pharmaceutics.

[36] Vandebriel R.J., De Jong W.H. (2012). A review of mammalian toxicity of ZnO nanoparticles. Nanotechnol. Sci. Appl..

[37] Naskar A., Bera S., Bhattacharya R., Saha P., Roy S.S., Sen T., Jana S. (2016). Synthesis, characterization and antibacterial activity of Ag incorporated ZnO–graphene nanocomposites. RSC Adv..

[38] Naskar A., Khan H., Sarkar R., Kumar S., Halder D., Jana S. (2018). Anti-biofilm activity and food packaging application of room temperature solution process based polyethylene glycol capped Ag-ZnO-graphene nanocomposite. Mater. Sci. Eng. C Mater. Biol. Appl..

[39] Naskar A., Cho H., Kim K.-S. (2022). Black phosphorus-based CuS nanoplatform: Near-infrared-responsive and reactive oxygen species-generating agent against environmental bacterial pathogens. J. Environ. Chem. Eng..

[40] Zhao Y., Jia Y., Xu J., Han L., He F., Jiang X. (2021). The antibacterial activities of MoS_2_ nanosheets towards multi-drug resistant bacteria. Chem. Commun..

[41] Zhao X., Chen M., Wang H., Xia L., Guo M., Jiang S., Wang Q., Li X., Yang X. (2020). Synergistic antibacterial activity of streptomycin sulfate loaded PEG-MoS_2_/rGO nanoflakes assisted with near-infrared. Mater. Sci. Eng. C Mater. Biol. Appl..

[42] Naskar A., Lee S., Lee Y., Kim S., Kim K.-S. (2020). A new nano-platform of erythromycin combined with Ag nano-particle ZnO nano-structure against methicillin-resistant *Staphylococcus aureus*. Pharmaceutics.

[43] Kim T.I., Kwon B., Yoon J., Park I.J., Bang G.S., Park Y., Seo Y.S., Choi S.Y. (2017). Antibacterial activities of graphene oxide–molybdenum disulfide nanocomposite films. ACS Appl. Mater. Interfaces.

[44] Kim T., Bak G., Lee J., Kim K.-S. (2015). Systematic analysis of the role of bacterial Hfq-interacting sRNAs in the response to antibiotics. J. Antimicrob. Chemother..

[45] Naskar A., Lee S., Kim K.-S. (2020). Antibacterial potential of Ni-doped zinc oxide nanostructure: Comparatively more effective against Gram-negative bacteria including multi-drug resistant strains. RSC Adv..

[46] Comfort N., Cai K., Bloomquist T.R., Strait M.D., Ferrante A.W., Baccarelli A.A. (2021). Nanoparticle tracking analysis for the quantification and size determination of extracellular vesicles. J. Vis. Exp..

[47] Qi Y., Wang N., Xu Q., Li H., Zhou P., Lu X., Zhao G. (2015). A green route to fabricate MoS_2_ nanosheets in water-ethanol-CO_2_. Chem. Commun..

[48] Li B., Jiang L., Li X., Ran P., Zuo P., Wang A., Qu L., Zhao Y., Cheng Z., Lu Y. (2017). Preparation of monolayer MoS_2_ quantum dots using temporally shaped femtosecond laser ablation of bulk MoS_2_ targets in water. Sci. Rep..

[49] Filipe V., Hawe A., Jiskoot W. (2010). Critical evaluation of nanoparticle tracking analysis (NTA) by NanoSight for the measurement of nanoparticles and protein aggregates. Pharm. Res..

[50] Wen T., Liu J., He W., Yang A., Xu H., Gu N. (2020). Nanomaterials and Reactive Oxygen Species (ROS). Nanotechnology in Regenerative Medicine and Drug Delivery Therapy.

[51] Bidkar A.P., Sanpui P., Ghosh S.S. (2019). Red blood cell-membrane-coated Poly(Lactic-*co*-glycolic Acid) nanoparticles for enhanced chemo- and hypoxia-activated therapy. ACS Appl. Bio Mater..

[52] Su J., Sun H., Meng Q., Zhang P., Yin Q., Li Y. (2017). Enhanced blood suspensibility and laser-activated tumor-specific drug release of theranostic mesoporous silica nanoparticles by functionalizing with erythrocyte membranes. Theranostics.

[53] Reshma V.G., Mohanan P.V. (2017). Cellular interactions of zinc oxide nanoparticles with human embryonic kidney (HEK 293) cells. Colloids Surf. B Biointerfaces.

