# Supplementary material for: A Nanocomposite with Extracellular Vesicles from Lactobacillus paracasei as a Bioinspired Nanoantibiotic Targeting Staphylococcus aureus
Source: Pharmaceutics. 2022 Oct 24;14(11):2273. doi: 10.3390/pharmaceutics14112273 (PMC9692410; doi:10.3390/pharmaceutics14112273)
Supplement: Supplementary file 1 [file pharmaceutics-14-02273-s001.zip › pharmaceutics-1963028-supplementary.pdf]

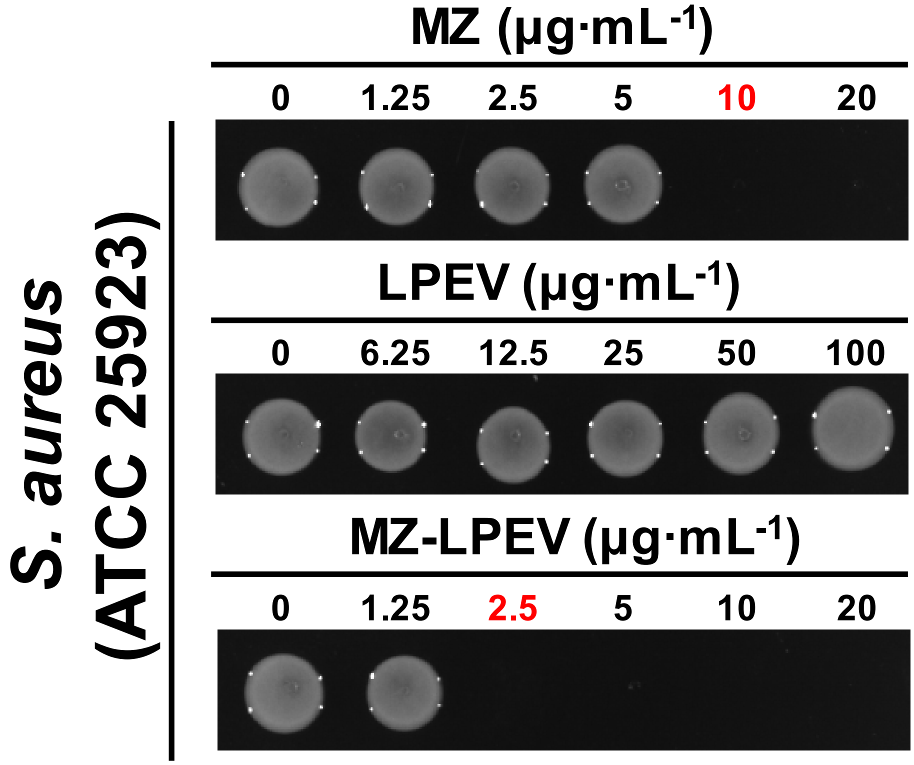

Figure S1. **Bactericidal activity of nanocomposites and LPEV.** Viability of *S. aureus* (ATCC 25923) after treatment with MZ, LPEV, and MZ-LPEV. The bacterial cultures are the same as those used for determining minimum inhibitory concentration (MIC) (**Table 2**), with 5 µL from each well being added to Luria-Bertani agar plates. The plates were further incubated at 37 °C for 16 h. The agar plate was imaged with ChemiDoc™ MP (Bio-Rad, CA, USA) and ImageLab™ Software (ver 5.02, Bio-Rad, CA, USA). Experiments were performed in triplicate. A representative plate is shown here.

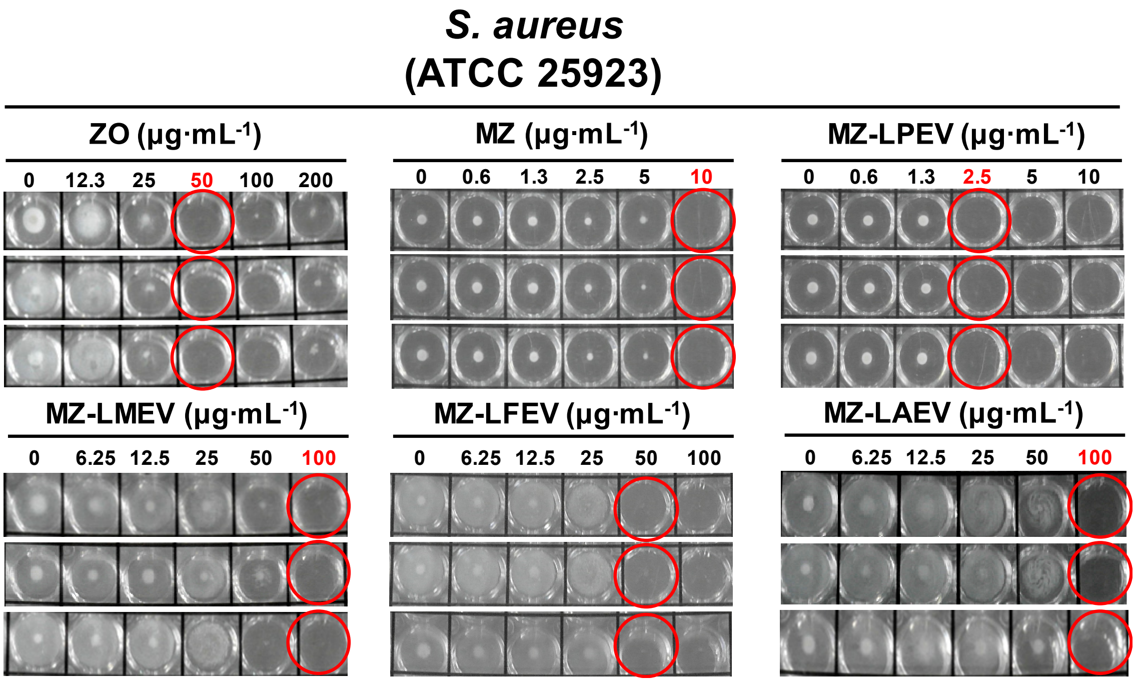

Figure S2. Antibacterial activity of nanomaterials. Data for determining minimum inhibitory concentrations (MICs) of ZO NP, MZ, and *Lactobacillus* strains-derived EV-coated MZ against *S. aureus* (ATCC 25923) are shown. Photographs of 96-well plates from triplicate experiments are shown. Red circles indicate the MIC values. The values are presented in **Table 2**.

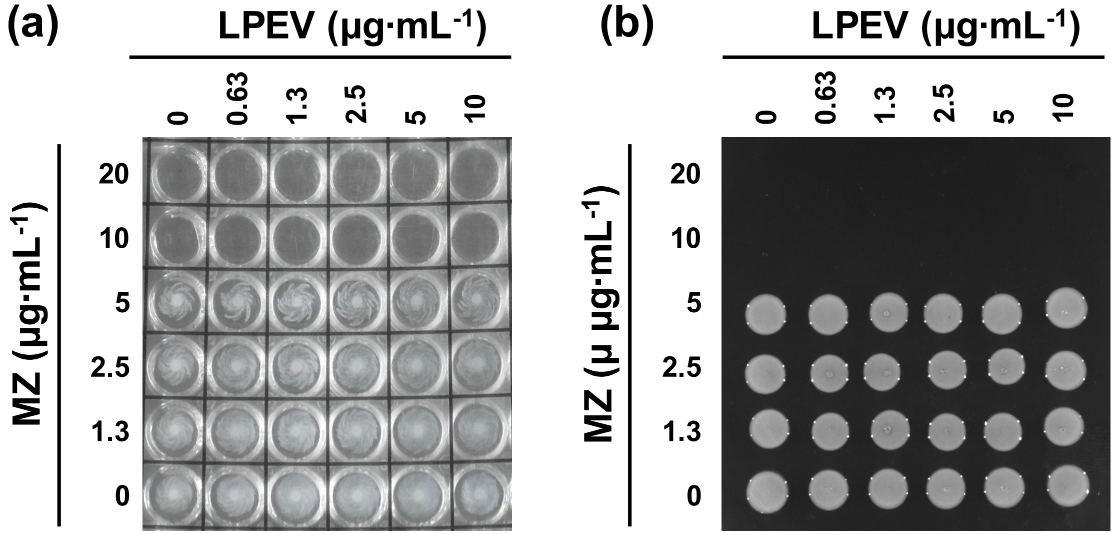

Figure S3. Evaluation of synergistic activity between MZ and LPEV. (a) Checkerboard assay. Result for *S. aureus* (ATCC 25923) was shown. **(b)** Viability assay. Aliquots of *S. aureus* (ATCC 25923) cells from (a) were spotted onto LB-agar plates and further incubated at 37 °C for 16 h. The agar plate was imaged with ChemiDoc™ MP (Bio-Rad, CA, USA) and ImageLab™ Software (ver 5.02, Bio-Rad, CA, USA). A representative replicate from triplicate experiments is shown.

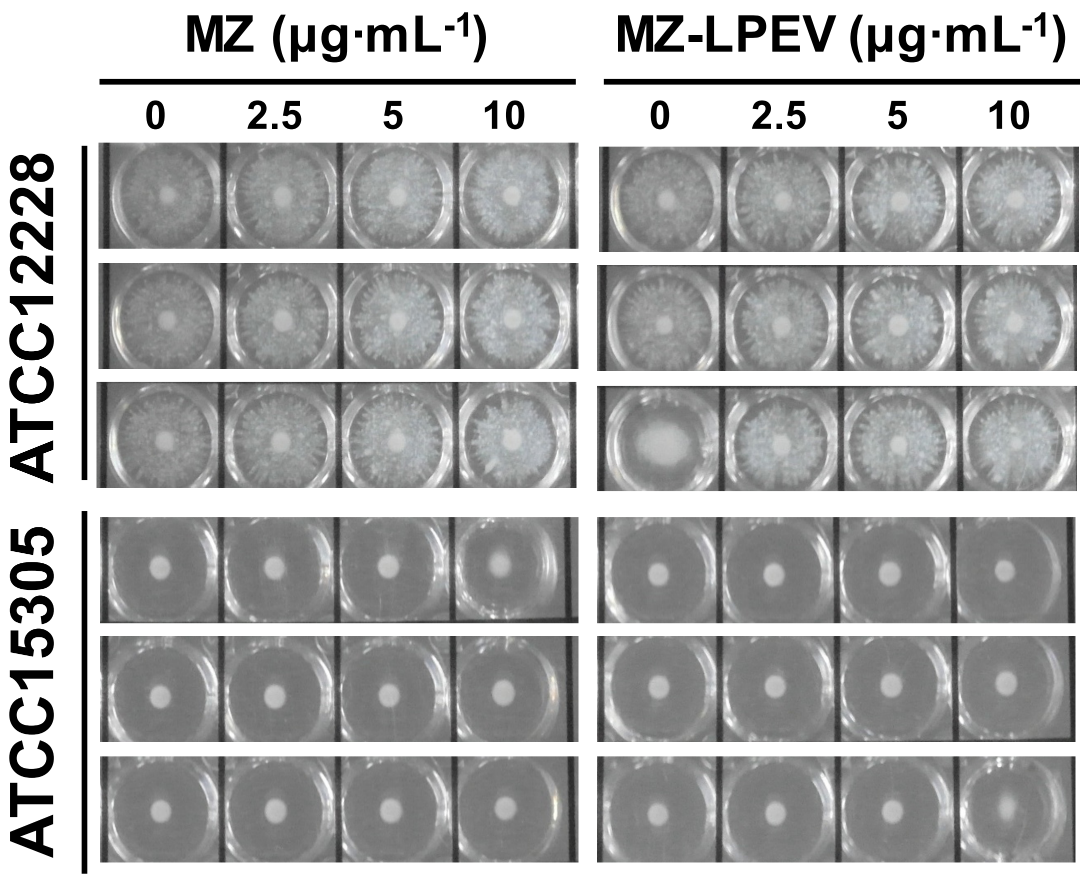

Figure S4. Antibacterial activity of nanocomposites and MZ-LPEV against Gram-positive bacteria. Minimum inhibitory concentrations (MICs) of MZ and MZ-LPEV against *S. epidermidis* (ATCC 12228) and *S. saprophyticus* (ATCC 15305). Results of MIC determination triplicate experiments in 96-well plates are shown.

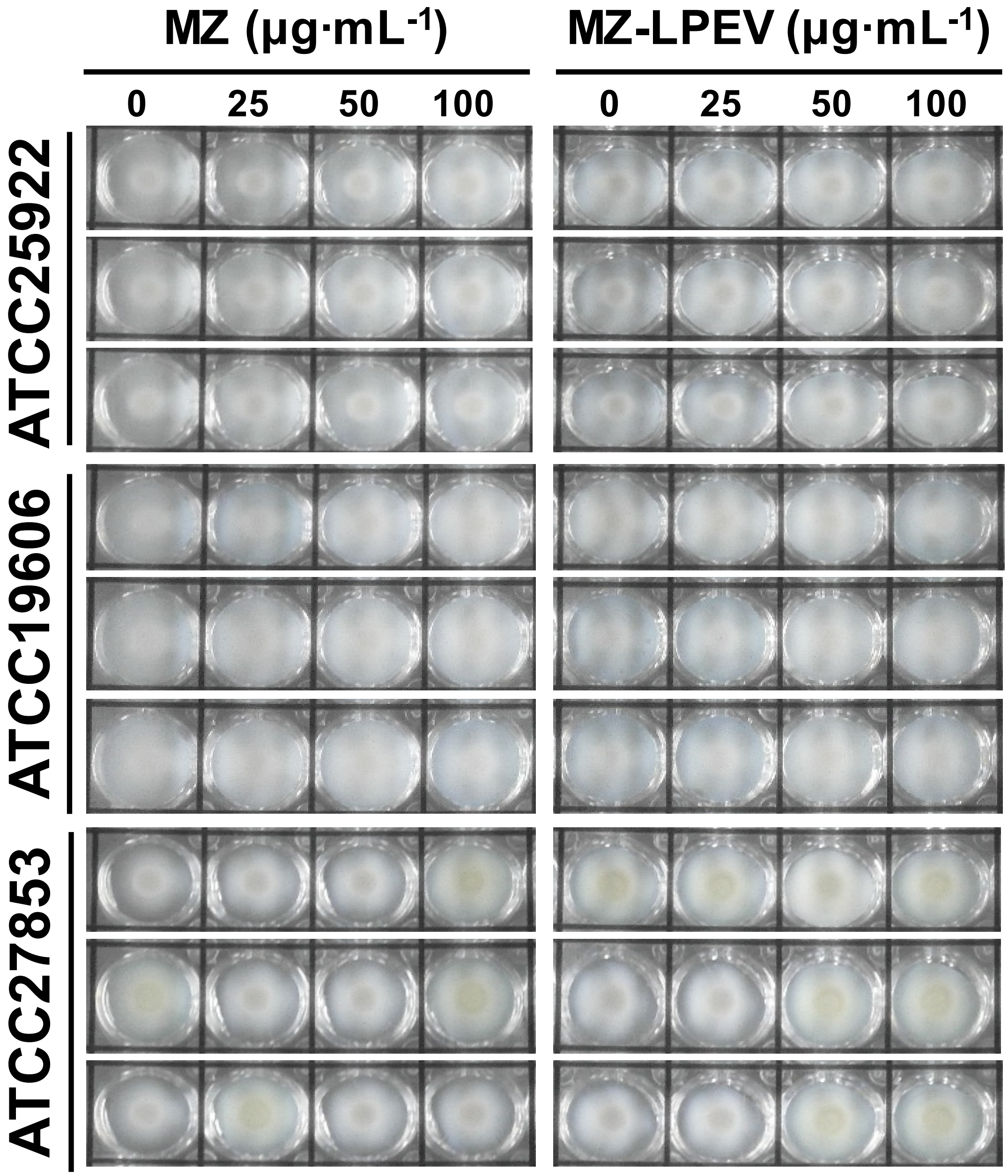

Figure S5. **Antibacterial activity of nanocomposites and MZ-LPEV against Gram-negative bacteria**. Minimum inhibitory concentrations (MICs) of MZ and MZ-LPEV against *A. baumannii* (ATCC 19606), *E. coli* (ATCC 25922), and *P. aeruginosa* (ATCC 27853*)*. The images of MIC results in 96-well plates from triplicate experiments are shown.
